# Exposure to Airborne Particles and Volatile Organic Compounds from Polyurethane Molding, Spray Painting, Lacquering, and Gluing in a Workshop

**DOI:** 10.3390/ijerph120403756

**Published:** 2015-04-02

**Authors:** Bjarke Mølgaard, Anna-Kaisa Viitanen, Anneli Kangas, Marika Huhtiniemi, Søren Thor Larsen, Esa Vanhala, Tareq Hussein, Brandon E. Boor, Kaarle Hämeri, Antti Joonas Koivisto

**Affiliations:** 1 Department of Physics, University of Helsinki, P.O. Box 48, FI-00014 Helsinki, Finland; E-Mails: tareq.hussein@helsinki.fi (T.H.); brandon.boor@helsinki.fi (B.E.B.); kaarle.hameri@helsinki.fi (K.H.); 2 Nanosafety Research Centre, Finnish Institute of Occupational Health, Topeliuksenkatu 41 a A, FI-00250 Helsinki, Finland; E-Mails: anna-kaisa.viitanen@ttl.fi (A.-K.V.); anneli.kangas@ttl.fi (A.K.); marika.huhtiniemi@ttl.fi (M.H.); esa.vanhala@ttl.fi (E.V.); joonas.koivisto@ttl.fi (A.J.K.); 3 National Research Centre for the Working Environment, Lersø Parkallé 105, Copenhagen DK-2100, Denmark; E-Mail: stl@nrcwe.dk; 4 Department of Physics, Faculty of Science, The University of Jordan, Amman, JO-11942, Jordan; 5 Department of Civil, Architectural, and Environmental Engineering, The University of Texas at Austin, Austin, TX 78712, USA

**Keywords:** ultrafine particles, particle size distribution, VOC, workplace aerosols, polyurethane molding, spray painting, occupational exposure, PID

## Abstract

Due to the health risk related to occupational air pollution exposure, we assessed concentrations and identified sources of particles and volatile organic compounds (VOCs) in a handcraft workshop producing fishing lures. The work processes in the site included polyurethane molding, spray painting, lacquering, and gluing. We measured total VOC (TVOC) concentrations and particle size distributions at three locations representing the various phases of the manufacturing and assembly process. The mean working-hour TVOC concentrations in three locations studied were 41, 37, and 24 ppm according to photo-ionization detector measurements. The mean working-hour particle number concentration varied between locations from 3000 to 36,000 cm^−3^. Analysis of temporal and spatial variations of TVOC concentrations revealed that there were at least four substantial VOC sources: spray gluing, mold-release agent spraying, continuous evaporation from various lacquer and paint containers, and either spray painting or lacquering (probably both). The mold-release agent spray was indirectly also a major source of ultrafine particles. The workers’ exposure can be reduced by improving the local exhaust ventilation at the known sources and by increasing the ventilation rate in the area with the continuous source.

## 1. Introduction

In small-scale workshops, it is common that various materials are processed in similar way as in industry, except at a smaller scale, and usually many of these processes are performed in the same space. Thus, workers are exposed to a complex mixture of pollutants; in most cases on a repeated daily basis. Control measures to reduce inhalation exposure may be ineffective or even absent [1], and high air pollutant concentrations may occur [1,2].

There are only few studies considering occupational airborne exposure levels in small-scale workshops [1,2,3], excluding studies in car repair shops. The exposure risk related to specific processes can be estimated qualitatively using industrial exposure studies, but the exposure levels are likely to be different. Considering risks related to specific industrial processes, polyurethane production involves isocyanate, which is a common cause of occupational asthma, and a few studies have investigated the isocyanate exposure of polyurethane molding workers [4,5]. The observations by Conner [6] suggest that also the mold-release agent can have a clear effect on the respiratory health of these workers. Spray painters are commonly exposed to volatile solvents [7], isocyanates [8,9] and to the pigment, which may contain harmful substances, such as chromate [10,11]. Other studies have shown that spray painters have increased risks for a variety of diseases [12,13,14]. Exposure to VOCs is also common among people working with glues [15,16] or lacquers [17].

The exposure studies among the groups of workers mentioned above have focused on specific expected pollutants, such as isocyanates. However, other pollutants may originate from the same sources, and pollutants may transform in the air due to chemical reactions or gas-to-particle conversion. In particular, there is a lack of knowledge on potential sources of fine particles in many occupational environments.

Inhalation exposure to airborne particulate matter (PM) and gaseous pollutants may, in general, increase the risk of a wide range of diseases, including: asthma, chronic obstructive pulmonary disease, cancer, atherosclerosis, arrhythmia, and stroke [18,19,20,21,22]. Also, studies focusing on occupational air pollution found that airborne PM exposure relates to various respiratory diseases [23]. There is also clear evidence that current occupational exposure limits for low-toxicity dusts [24] are not sufficiently low to prevent adverse health effects of long term PM exposure [25,26]. Moreover, the PM exposure metrics associated to health effects are under discussion [27,28,29]. This is partly due to the evidence that ultrafine particles (diameter < 100 nm), which only contribute little to the regulated mass concentrations, have adverse health effects [30,31,32,33,34].

In order to understand concentration levels and to identify sources in a ~500 m^2^ handcraft workshop manufacturing fishing lures, we measured PM and total volatile organic compound (TVOC) concentrations. The main processes were spray painting with airbrushes, polyurethane molding, gluing, and lacquering. TVOC and size resolved particle concentrations were measured continuously for six days using instruments with time resolutions ranging from 1 s to 5 min. Measurements were made parallel from outdoors and indoors, and indoor PM and TVOC concentrations were linked with work activity so that we could identify emission sources [35,36].

## 2. Methods

Particle and TVOC concentrations were measured continuously from Wednesday to Monday (13–18 November 2013). Temperatures measured by the Finnish Meteorological Institute at the nearest weather station ranged from −1 °C to 8 °C.

### 2.1. Work Environment and Processes

**Figure 1:**
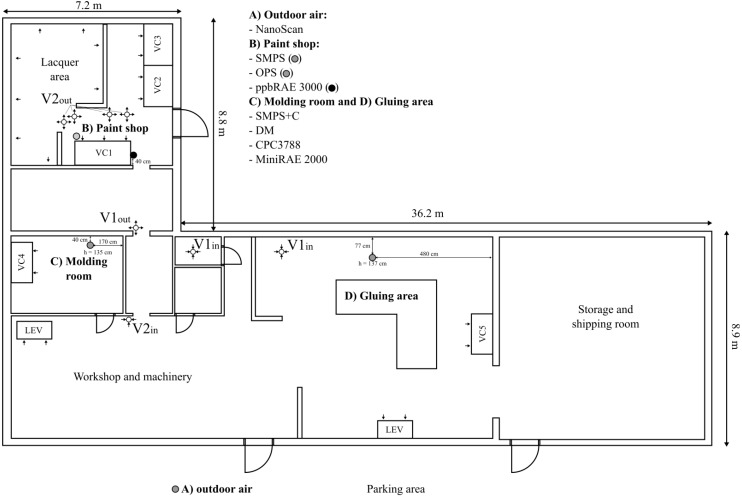
Layout of the workshop. The gray dots and the black dot denote measurement locations. The small arrows denote mechanically induced airflows. LEV and VC are abbreviations for local exhaust ventilation and ventilated cupboard, respectively.

The workshop was located at a rural area in southern Finland and employed approximately 10 persons. In this study, the measurements were carried out in three main working areas: molding room, paint shop, and gluing area (Figure 1). The building ventilation was supported with mechanical exhaust ventilation systems (see arrows in Figure 1). The replacement air was drawn from outdoor air through the building shell and through the door at the paint shop which was open during most of the working hours. The indoor air was also mechanically circulated inside the workshop (see Figure 1; V1_in_–V1_out_ and V2_in_–V2_out_). The doors between different working areas were open so the air could mix inside the whole workshop. The working hours were registered separately for each of these three locations. In our analysis we defined non-working hours as all times without working activities within the last 90 min anywhere in the workshop.

#### 2.1.1. Molding Room

**Figure 2:**
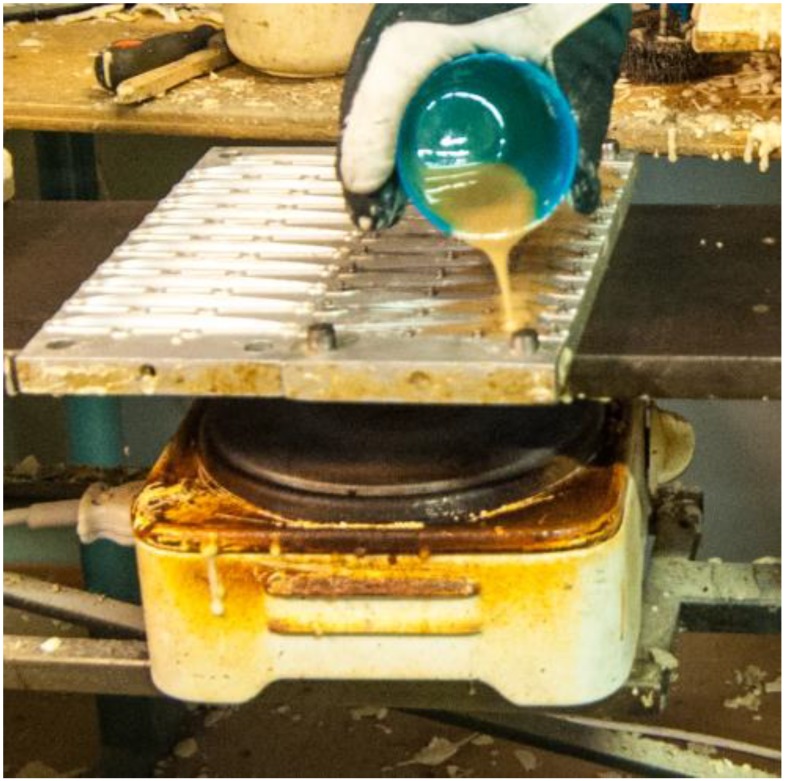
Molding mass poured onto a mold. The hotplate is seen underneath.

In the molding room, a hot plate was turned on around 7:00 and the molding work was carried out by one person starting from around 9:00. A polyurethane mold-release agent (Acmos 36-7120, Lagotech Ab., Mölnlycke, Sweden) was sprayed and brushed on a metal mold. This mold-releasing agent contains 86%–95% C_9_–C_12_ alkanes. Then a molding mass comprising an isocyanate (30.5%–32.5% w/w methylene diphenyl diisocyanate MDI, Desmodur 44 V 20 L, Bayer MaterialScience AG, Leverkusen, Germany) and a polyether polyol (Baydur I-300-A-45/W, BaySystems NorthernEurope A/S, Otterup, Denmark) was mixed 20 s with a power drill and poured on the mold (Figure 2). The mold was closed with a screw clamp and left for approximately 10 min during which the isocyanate and the polyether polyol reacted to form polyurethane. Occasionally the mold was washed with Bomix Mould Cleaner 60/692 (Bomix, Teglte, Germany).

#### 2.1.2. Paint Shop

Painting primarily occurred between 6:00–16:00 when up to three workers were working at the same time. Occasionally, there was work activity during the evening and on Sunday at 15:10–15:50. The lures were handled mainly in 21 piece combs. The lures were first dipped into a pool of lacquer (probionate, Albis Cellidor, Hamburg, Germany) and then dried. This was repeated 5 to 6 times after which a primer (NAV-paint, Lakkavalmiste Oy, Tampere, Finland) was applied. Painting was made with an airbrush (Badger Crescendo 175, Franklin Park, IL, USA) in ventilated cupboards (see VC1 to VC3 in Figure 1). The lures were dipped into lacquer (Cab-Coatings Finland, Tampere, Finland) while different layers of paint were applied.

#### 2.1.3. Gluing Area

Gluing typically occurred between 7:00–15:30 and was performed by hand (UHUallplast, UHU GmbH, Bühl/Baden, Germany), mainly, and with spray glue (Vertex K 810, Kiilto Oy mixed with thinner for polychloroprene contact adhesives, Kiilto Oy, Tampere, Finland). In the gluing area, other processes such as small-scale wood sawing and grinding also occurred. There were usually three to four workers present.

### 2.2. Sampling of Airborne Particles

A variety of particle sampling instruments was used to concurrently monitor airborne particle concentrations and size distributions at different locations in the shop (along with one outdoor location). The instruments allowed us to study aerosol particles from less than 10 nm to several µm in diameter. The particles were sampled through electrically conductive sampling lines or without sampling lines. Diffusional losses for the sampling lines were corrected for the mobility particle sizers according to Cheng [37]. Sampling line inlets heights varied from 110 to 170 cm and their locations are marked in Figure 1.

A condensation particle counter (CPC, TSI Model 3788, TSI Inc., Shoreview, NM, USA, *Q_s,3788_* = 1.5 L∙min^−1^) measured total particle number concentration. Detection size range was approximately from 2.5 nm to 3 µm and the sampling time resolution was 1 second.

A portable condensation particle counter (TSI Model 3007, TSI Inc., *Q_s,3007_* = 0.74 L∙min^−1^) measured total particle number concentration. Detection size range was approximately from 7 nm to >1 µm and the time resolution was 1 s.

A scanning mobility particle sizer (SMPS) consisted of a classifier (TSI Classifier model 3080, pre-impactor with a 710 nm *D_50_* cut size, and a 10 μCi ^85^Kr neutralizer, TSI model 3077A, *Q_classifier inlet_* = 1.45 L∙min^−1^), differential mobility analyzer (DMA, Hauke-type, length 28 cm, inner diameter 2.5 cm and outer diameter 3.3 cm, sheath air flow 15 L∙min^−1^), and a condensation particle counter (CPC, TSI model 3776) measured particle number size distributions in 101 channels from 8.5 to 310 nm. The SMPS scan time was 45 s with a 15 s retrace time.

An aerosol mobility spectrometer (NanoScan, TSI Model 3910, *Q_s,NanoScan_* = 0.66 L∙min^−1^, inlet cyclone cut-size was approximately 500 nm) measured outdoor particle size distributions in 13 channels from 10 to 420 nm. The NanoScan scan time was 45 s with a 15 s retrace time.

A sequential mobility particle sizer and counter (SMPS + C, Grimm Series 5400, Ainring, Germany, *Q_s,SMPS_ + C* = 0.31 L∙min^−1^) with a medium differential mobility analyzer (“Vienna” type U-DMA) measured particle number size distributions in 44 channels from 5.5 to 350.4 nm. The SMPS + C scan time was 110 s with a 10 s retrace time.

An optical particle sizer (OPS, TSI model 3330, *Q_s,OPS_* = 1.02 L∙min^−1^) measured particle number distributions in 14 channels from 300 nm to 6 μm with a time resolution of 60 seconds. Coarse particle losses in the sampling line were corrected for using experimentally determined values for the size-resolved penetration efficiency.

A Dust Monitor (DM, Grimm Aerosoltechnik Model 1109, Ainring, Germany, *Q_s,DM_* = 1.21 L∙min^−1^ using the instrument default settings which assume particles having refractive index of 1.59 + 0*i*, density of 2600 kg∙m^−3^, and spherical shape) measured particle number size distributions in 16 channels from 260 nm to 31 μm with a time resolution of 60 s.

### 2.3. Combining Mobility and Optical Particle Number Size Distributions

The mobility and optical particle number size distributions measured by the SMPS and OPS, and respectively SMPS + C and DM were combined to form wide size-range d*N*/dLog(*D_p_*) number size distributions. To make this combination it was assumed that a particle’s mobility diameter and optical diameter were the same. The particle size distributions were based on the SMPS or SMPS + C for diameters up to 300 nm, and on OPS or DM for diameters above 300 nm. For the paint shop the combined size distribution based on SMPS and OPS measurements ranged from 8.5 nm to 9 μm. For the molding room and gluing area the size distributions ranged from 5.5 nm to 31 μm, and were based on SMPS + C and DM measurements.

### 2.4. Comparability between Instruments

In this study particle concentrations were measured with different particle instruments in different locations. It is well known that aerosol instruments concentration readings depend on, for example, the measurement size range, detection technique, data inversion, and particle penetration to the detector [38,39,40,41]. The comparability of different instruments used in this study was therefore evaluated during the measurement campaign. For this purpose, the portable CPC (3007) was utilized as a reference instrument because its properties are well known [42].

Instruments with measurement size ranges similar to the portable CPC (3007) showed similar concentrations, except for the SMPS + C which gave concentrations that were on average more than three times higher. Moreover, the SMPS + C was used in parallel with the CPC (3788) and consistently showed at least twice as high concentrations. Also, Asbach *et al.* [38] found that the SMPS + C showed much higher concentrations than the portable CPC (3007) and two SMPSs from TSI. In the concentration range observed in this study, the portable CPC (3007) has earlier been found to be reliable [42], so it is assumed here that the discrepancies are caused by heavy overestimation of the concentration by the SMPS + C. The overestimation is likely to depend somewhat on the particle size distribution. Due to this overestimation, all of the reported particle number concentrations in the molding room and the gluing area were based on the CPC data, and the SMPS + C data was only used for reporting the measured size distributions.

### 2.5. VOC Measurements

Concentrations of TVOC were measured with two photoionization detectors (PIDs, MiniRAE 2000 and ppbRAE 3000, RAE Systems Inc., San Jose, CA, USA). The PIDs were calibrated using isobutylene. TVOC concentrations can be measured in other ways as well, and because different types of instruments are sensitive to different compounds, the concentration obtained here may not be comparable to concentrations measured with other methods.

### 2.6. Particle Emissions in Molding Room

Typically, particle emissions occurred in short (up to a few minutes) bursts, and for a few size sections we estimated the numbers of particles released. To estimate the particle emission in each burst we simply multiplied the particle number concentration increase by the volume of the molding room. This procedure only gives rough estimates, because it is based on two questionable assumptions: no loss of particles between the start of the emission and the time when the maximum concentration is reached, and the air remains well-mixed at this time.

### 2.7. Electron Microscopy

Particle samples from the workplace air were collected onto holey carbon film-coated copper grids of 200-mesh (SPI, West Chester, PA, USA). The grids were attached on polycarbonate filter fixed on air sample cassettes (SKC Inc., Eighty Four, PA, USA, inlet diameter 1/8 inch and filter diameter 25 mm) and the sampling flow rate was 6 L∙min^−1^. Transmission electron microscopy (TEM, Jeol, JEM 1220, Tokyo, Japan) was used for imaging of particles and elemental analysis of single particles was conducted with an EDX-analyzer (Noran System Six, Thermo Fisher Scientific Inc., Waltham, MA, USA) attached to the TEM. The TEM was operated in scanning transmission electron microscopic mode (STEM) at 100 kV.

## 3. Results and Discussion

### 3.1. Particle Concentrations and Sources

**Figure 3:**
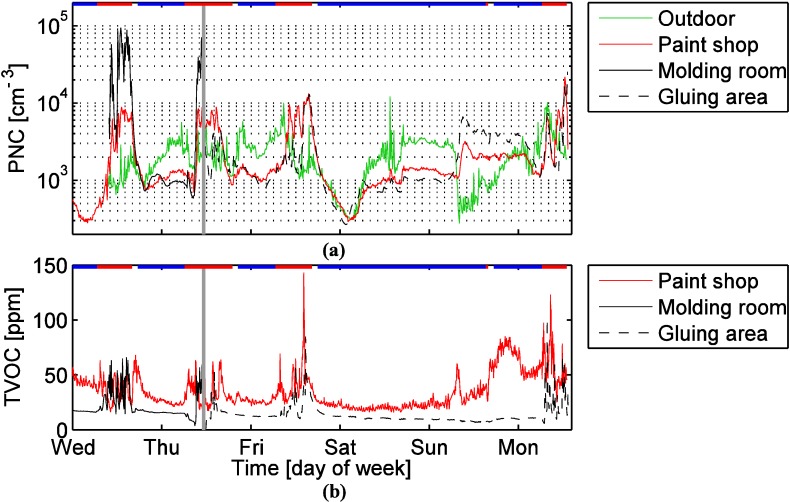
(**a**) Particle number concentrations at 10 minute resolution. (**b**) TVOC concentrations at five minute resolution. The horizontal red lines show the working hours and the horizontal blue lines show non-working hours. The vertical gray line shows when the instruments were moved from the molding room to gluing area.

Figure 3 shows that during working hours, the particle number concentrations were highest in the molding room, but during non-working hours the concentrations were similar in the molding room and the paint shop. The paint shop and the gluing area had very similar particle number concentrations most of the time. The mean working hour particle number concentration was 36,000 cm^−3^ in the molding room. This is a relatively high concentration, far above the working hour concentrations measured in the other locations (4500 cm^−3^ in the paint shop, 3300 cm^−3^ in the gluing area, 2500 cm^−3^ outdoors), but it is far from being exceptional in occupational settings [2,43,44]. The outdoor mean was calculated for the working hours in the paint shop.

The source which caused the elevated concentration in the molding room was identified using the portable CPC. Before a mold was filled the mold-release agent was sprayed onto it. The mold was in front of VC4 (Figure 1) on a table, which was heated by a hot plate placed under it (Figure 2). Usually some of the spray hit the warm table, and then new particles appeared. During our measurements in the molding room, this caused 17 major bursts of 10^12^–10^13^ particles; typically with a count median diameter of about 10 nm and a geometric standard deviation of 1.7. Because of high diffusivity these particles are likely to deposit mainly in upper airways or alveolar region when inhaled. Most likely, spray droplets evaporated at the warm surface, and then the vapors nucleated into new particles. It is also possible that the nucleating compounds were formed via chemical reactions.

**Figure 4:**
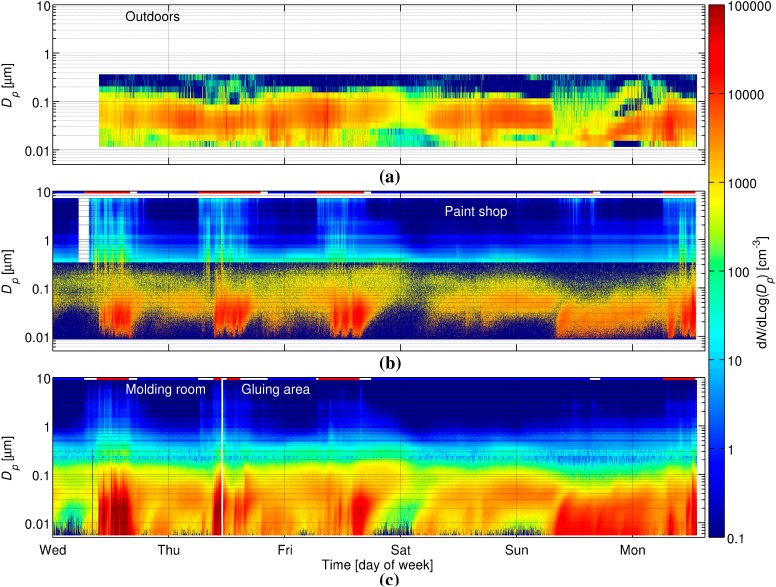
Particle size distribution time series. (**a**) Outdoors measured by NanoScan, (**b**) in the paint shop measured by SMPS and OPS, and (**c**) in the molding room (Tuesday 16:14–Thursday 11:04) and the gluing area (Thursday 11:07–Monday 13:20) measured by SMPS + C and DM). In figure (**b**) and (**c**) horizontal red lines shows the working hours in the respective locations and blue lines non-working hours.

**Figure 5:**
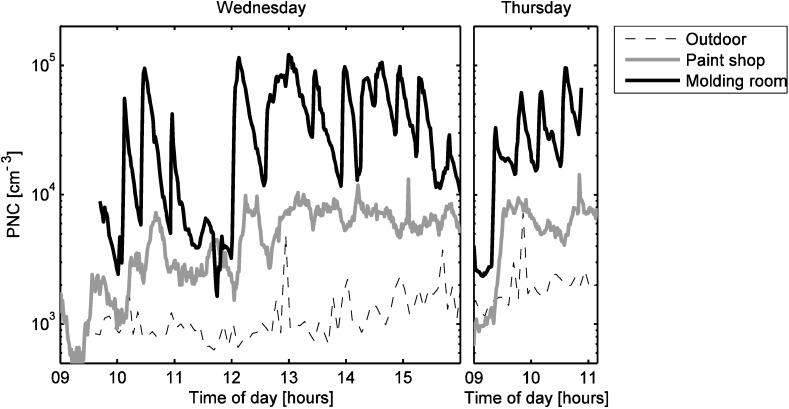
Particle number concentrations in the molding room, in the paint shop, and outdoors.

Some of these particles were transported to the paint shop where they caused the concentration to increase substantially. We can conclude this from Figure 3, Figure 4 and Figure 5. Figure 3 and Figure 4 show that the concentration did not increase much in the paint shop before the particles were formed in molding room, although the work was started in the paint shop a few hours earlier. Figure 5 shows that when the concentration peaked in the molding room, then usually a smaller peak appeared in the paint shop within 15 min. Thus, most of the ultrafine particles in the paint shop originate from the molding room. The inter-zonal transport of particles from the molding room to the paint shop is enhanced by one of the ventilation ducts (Figure 1). We have no simultaneous measurements in the gluing area and the molding room, but particles transported from the molding room are the most likely reason for the particle number concentration elevation during working hours in the gluing area as well.

Not all of the particles formed indoors can be related solely to working processes. For example, on Sunday, concentrations of ultrafine particles were elevated (Figure 4) even though workers were not present for most of the day. It seems that indoor secondary particle formation occurred more or less continuously for about 20 h starting from about 7:40 Sunday morning. At this time the outdoor concentration measured by the NanoScan suddenly decreased, but it is unclear whether this decrease is real or caused by some instrumental error (e.g., due to low temperatures). Coarse particle contribute very little to the particle number concentrations discussed above, but Figure 4 reveals that the concentration of coarse particles was elevated during working hours, especially in the paint shop. Spray painting was probably the main source of coarse particles in the paint shop.

### 3.2. TVOC Concentrations

During working hours the TVOC concentration was generally higher in the paint shop (41 ppm on average) and the molding room (37 ppm) than in the gluing area (24 ppm, see also Figure 3). During non-working hours the VOC concentration was clearly reduced in the molding room (17 ppm) and the gluing area (11 ppm), but not so much in the paint shop (32 ppm). These concentrations indicate that there were continuous emissions keeping the concentration high in the paint shop. Evaporation of VOCs from the lacquer pools and other containers of lacquer and paint caused at least part of these emissions. During working hours, additional VOCs are expected to originate from the spray painting and lacquering; both during these processes and when the lures dried up afterwards. These emissions explain that the TVOC concentration was somewhat higher during the working hours. The fluctuation of the concentration is probably related to fluctuation in sources and changes in the ventilation rate which varied depending on the use of the VCs’. On Wednesday, Thursday, and Friday the paint shop TVOC concentration increased around 11:00 and remained elevated for about half an hour. The most likely reason is that at that time the workers turned the ventilation in the ventilated cupboards (VC1 to VC3) off and went for lunch.

**Figure 6:**
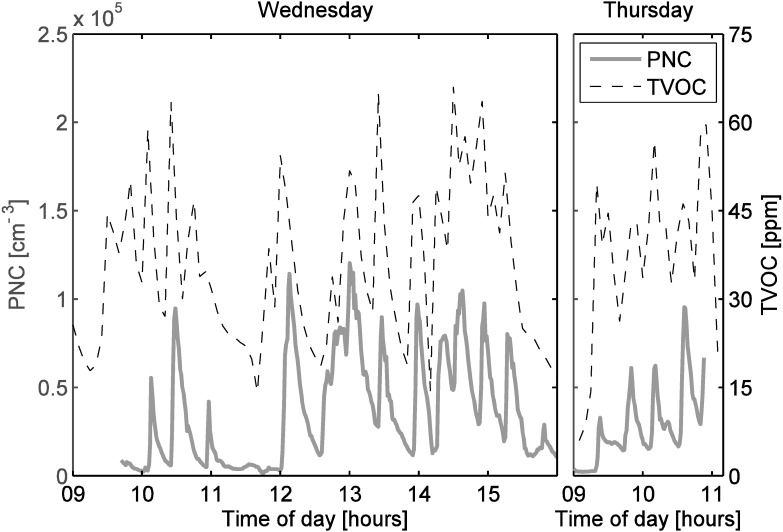
Particle number concentrations and TVOC in the molding room. The two subfigures have same concentration scales both for particle number concentration and TVOC.

Also in the molding room we observed fluctuating TVOC concentrations during working hours. In fact, almost all substantial increases in the particle number concentration were accompanied by clear increases in the TVOC concentration (Figure 6). Only the particle number concentration peak at 10:55 was an exception: at this time the TVOC concentration increase was small. Despite this exception it seems evident that the spray, which was the indirect source of the particles, also caused substantial VOC emissions.

On Thursday spray gluing was reported for the gluing area in the time interval 13:18–14:50 which was the most likely reason for the increased TVOC concentration observed in Figure 3. The elevated concentration on Friday and Monday may to some extend be explained by VOCs transported from the paint shop or molding room, but the peaks are probably caused by local emissions from gluing.

The TVOC concentrations in the workshop were high compared to TVOC concentrations measured in other working places. Stefaniak *et al.* [45] measured TVOCs in two photocopy centers, and obtained daily averages ranging from 0.2 to 6.2 ppm. Likewise, Sarkhosh *et al.* [46] obtained mean concentrations below 0.3 ppm in four photocopy centers. Bello *et al.* [47,48] observed mean TVOC concentrations ranging from 0.02 to 6.5 ppm during cleaning activities. Goldin *et al.* [49] investigated the air quality in 21 nail salons. The mean TVOC concentrations in these salons ranged from 0.1 to 38 ppm with a median of 4.8 ppm. Preller *et al.* [50] observed higher concentrations though. They measured during spraying activities in different companies and found a mean concentration of 78 ppm. These studies all used PID for their measurements and most calibrated the PIDs with isobutylene, but a few omitted this information. The study by Preller *et al.* [50] differed by using measurements of the composition to correct the PID measurement for different sensitivities to different VOCs. Thereby they obtained a more accurate measure of the TVOC concentration.

### 3.3. Electron Microscopy

**Figure 7:**
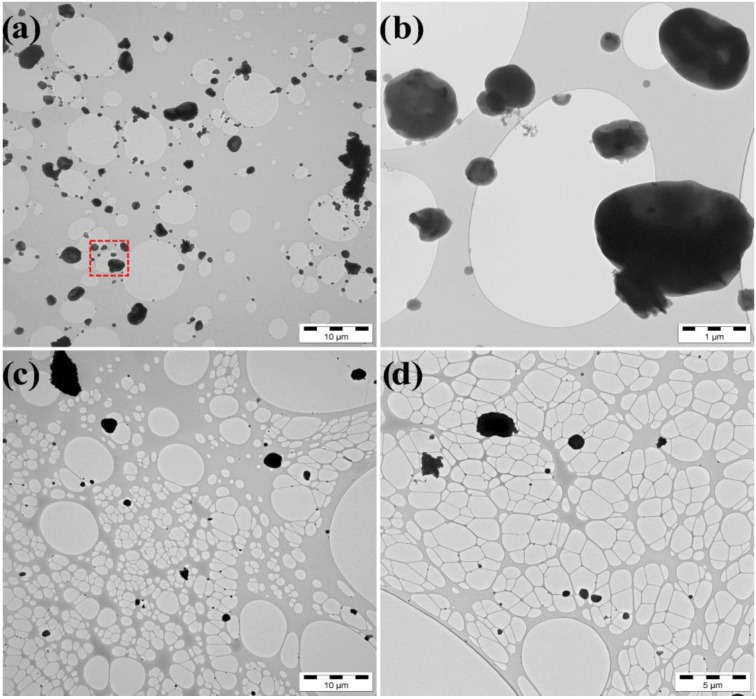
Electron micrographs of particles sampled from (**a**) the paint shop where dashed rectangle is magnified in (**b**), (**c**) the molding room, and (**d**) the gluing area.

Electron microscopy showed that the particles were close to spherical and had diameters above 200 nm (Figure 7). The smaller particles (see Figure 4) were most likely evaporated in electron microscope and therefore not detected in significant numbers. In the paint shop, the particles consisted mainly of C and some Cl, and they were up to several µm in diameter (Figure 7a,b). A fraction of these particles contained elements, such as Bi, V, Ti, and Ba, which can be attributed to pigments, which originated from the spray painting. In the molding room, the particles consisted mainly of C and often also some S, and approximately 25% included also Cr (Figure 7c). In the gluing area, particles consisted mainly of C and often also some Cl and approximately 25 % included also Cr. The sample also contained spherical NaCl-particles including small amounts of K, Ca, and S (Figure 7d). Soot particles and spherical particles containing mostly S originate from traffic outdoors and were present in all samples.

### 3.4. Representativeness of Measurements and Limitations of Study

The processes that were observed over the course of the sampling period are typical of the activities at the shop on a weekly basis. Therefore, we would expect the workers to be exposed to similar concentrations of particles and TVOC on a repeated, daily basis. The measured particle number concentrations are in general representative for the particle number concentrations in the respective rooms. In the paint shop and gluing area the emitted number of particles was not substantial, so the gradients must have been small, and thus the measurements in these places are representative. In the molding room measurements with the portable CPC showed that the gradient was small except during and briefly after the spraying. In this room the VOCs originated from the same source, so we expect the same to hold true for VOC concentrations. In the gluing area the TVOC sources were small most of the time, so we expect that the gradient was small as well. However, during the spray gluing the measurements were probably not so representative. In the paint shop there were substantial VOC sources and the TVOC concentration gradients may have been considerable. However, the measurements are representative enough to conclude that the TVOC concentration was substantially higher in the paint shop than in the other locations. In the exposure assessment, it must be kept in mind that stationary measurements may underestimate [51,52] or overestimate [53] the personal exposure concentrations.

We had no quantitative information about ventilation rates or indoor airflows, so only little information about the migration of pollutants could be obtained. The PID measurements gave no information about which VOCs were present. Such information would have been useful for pointing out the most important VOC sources in the paint shop, and for estimating health hazards.

### 3.5. Exposure Reduction

Efforts to reduce concentrations and control source emissions are recommended. In the molding room we found one strong source of particles and VOCs. The molds were on a table in front of the ventilated cupboard VC4 (Figure 1), but there was no local exhaust ventilation at the source. We recommend that local exhaust ventilation is adopted either by modifying VC4, so that the table with the molds is inside of it, or by installing a movable hood. In the paint shop the TVOC concentration was high due to substantial sources. One way to reduce the concentrations is to increase the ventilation rate, use air tight lids on lacquer pools, and using ventilated closet for lacquer and paint canisters. Because the air was mixed between the areas, removal of pollutants in one place would improve the air quality in the whole workshop.

### 3.6. Potential Health Hazards

The VOCs measured in the present study derive at least in part from the specific chemical substances used for the manufacturing of fishing lures. The different VOCs have different toxicities, and include hydrocarbons (C_9_–C_12_ alkanes) from the polyurethane mold release substance. Alkanes are in general well tolerated by the organism which is reflected by the relatively high occupational exposure limits (OEL) for these substances. For instance, the OELs for nonane are 200 ppm (8 h time-weighted average) and 250 ppm (15 min, short term exposure limit) [54]. During the molding process, workers handle a mixture containing MDI. MDI is, along with other isocyanates, a well-known inducer of occupational asthma [55]. This potential to induce occupational asthma has led to low OELs in the range 5 ppb (8 h time-weighted average) and 20 ppb (10 min) [56]. The very low vapor pressure of MDI (boiling point 314 °C) inhibits development of high concentrations in the gas phase. In the present case, MDI was however heated which has likely increased the emission rate and gas concentration, but a specific chemical analysis for MDI is needed in order to clarify whether the OEL has been exceeded in the specific case.

Based on the product data sheets, the ultrafine particles emitted in the molding room are unlikely to contain anything more harmful than MDI. As a worst case scenario, assuming that these ultrafine particles consisted purely of MDI, their mass concentration was about 0.4 μg/m^3^, corresponding to a concentration of 0.04 ppb. This is far below the OEL. However, the number concentration of 36,000 cm^−3^ is high, well above typical ambient urban conditions, and there are indications that the high number (or surface area) in itself poses a health risk independent of the toxicity of the material [57].

## 4. Conclusions

We measured pollutant concentrations in a fishing lure workshop located in a rural area in Finland. The mean working-hour TVOC concentrations ranged from 24 to 41 ppm. The highest concentration was measured in the paint shop and was due to spray painting, lacquering, and continuous evaporation from lacquer pools and other lacquer and paint containers. In the gluing area, spray gluing was a VOC source. In the molding room the mold-release agent spray, which was applied about twice per hour, caused substantial VOC emissions, and the average working hour TVOC concentration was 37 ppm. This spray was indirectly also the main source of ultrafine particles, which are likely to deposit when inhaled. When this spray was applied to the molds, part of it hit the warm table surface, and then up to $10^{13}$ particles were emitted. This caused a mean working-hour concentration of 36,000 cm^−3^ in the molding room, while elsewhere the mean particle number concentration was below 5000 cm^−3^. A substantial improvement of the local exhaust ventilation near known sources and a general increase of the ventilation rate are recommended.
